# Ventilator-induced lung injury in rat models: are they all equal in the race?

**DOI:** 10.1186/s42826-025-00240-y

**Published:** 2025-05-19

**Authors:** Jon Petur Joelsson, Sigurbergur Karason

**Affiliations:** 1 University of Iceland, Reykjavik, Iceland; 2 Landspitali-University Hospital, Reykjavik, Iceland

**Keywords:** VILI, ARDS, Mechanical ventilation, Rat models

## Abstract

Risk of ventilator-induced lung injury (VILI) is an inevitable and precarious accompaniment of ventilator treatment in critically ill patients worldwide. It can both instigate and aggravate acute respiratory distress syndrome (ARDS) where the only prevention or treatment so far has been empirical approach of what is considered to be lung protective ventilator settings in an attempt to shield the lung tissues against the mechanical stress that unavoidably follows ventilator treatment. The weakened state of the patients limits clinical drug research and pushes for drug discovery in animal models. Mice and rats are often the choice of small animal model, representing about 95% of all laboratory animal studies, as their physiology can mimic that which is found in humans. Mice have been a more popular choice for ventilator studies but due to technical issues, there is some advantage gained in using rats as they are substantially larger. Inducing VILI and ARDS in these models can prove challenging and often the acute nature of the injury used to produce similar tissue damage as in humans does not necessarily fully reflect clinical reality. The aim of this review was to analyse and summarize methods of recent publications in the field, describing what approaches have been utilized to simulate these conditions, possibly identifying a common track enabling comparison of results between studies. However, the study shows a high variety of methods employed by researchers causing comparisons of results difficult and perhaps implying that a more standardized approach should be used.

## Background

Mechanical ventilation (MV) is a necessity in intensive care units worldwide. MV abates the work of breathing for patients who are too sick to ensure sufficient gas exchange for themselves. An inherent risk of ventilating patients manifests in ventilator-induced lung injury (VILI), a common complex adverse consequence of MV. The main causes of VILI comprise atelectrauma, baro- volutrauma, and oxygen toxicity leading to biotrauma. Atelectrauma occurs when the repeated opening, during inspiration, and collapse of the alveoli, during expiration, results in shear stress with ensuing damage. Baro- volutrauma are two sides of the same coin and is caused by too much pressure or volume of air applied to the lungs causing overdistension. It is often a consequence of collapsed regions of the lungs, where the open areas receive too much pressure or large distending volume of air. Use of high inspiratory fraction of oxygen may lead to creation of free radicals leading to tissue injury. Biotrauma is the resulting response to these injurious mechanisms, reflected in the release of inflammatory mediators from nearby immune cells and other local cells, causing further tissue injury in the lungs, and may be carried with circulation to distant organs causing multi organ failure [1]. Acute respiratory distress syndrome (ARDS) is another unfortunate affliction, caused by either direct or indirect injury to the lungs, that calls for MV. ARDS is a complex syndrome with a high mortality rate, characterized by insufficient oxygenation and loss of compliance in lungs. Barrier failure of the epithelial and endothelial border ensues along with diffuse alveolar damage [2]. Patients with serious ARDS always require MV, because of insufficient gas exchange, that may call for high ventilatory settings, and the weakened state of the lungs make them susceptible to VILI. Avoidance of VILI has currently been focused on lung protective ventilation using positive end expiratory pressure (PEEP) to keep the alveoli from collapsing at end of expiration and avoiding use of high peak inspiratory pressures to prevent overdistension. However, these are only empirical recommendations, and the lungs or its alveoli are heterogeneously injured so there is no single ventilator setting that can fully avoid the risk of VILI [3]. No drug therapy is currently being used to mitigate the effects of VILI, although this is a hot topic of research. As the patients suffering from VILI/ARDS are highly vulnerable, animal models are often used to mimic the diseased state and for testing various therapeutics. We previously reviewed mouse models of VILI, which are by far the most popular choice [4]. Another popular choice is the rat model, but these two animals make up 95% of animal research [5].

The aim of this paper is to summarize methods that researchers have used to provoke ventilator-induced lung injury in rat models the last few years. For this purpose we typed into PubMed “Ventilator induced lung injury rat model” and summarized papers from 2016 to 2024 [6–68]. We focused only on publications where rats were mechanically ventilated.

## Main text

**Fig. 1:**
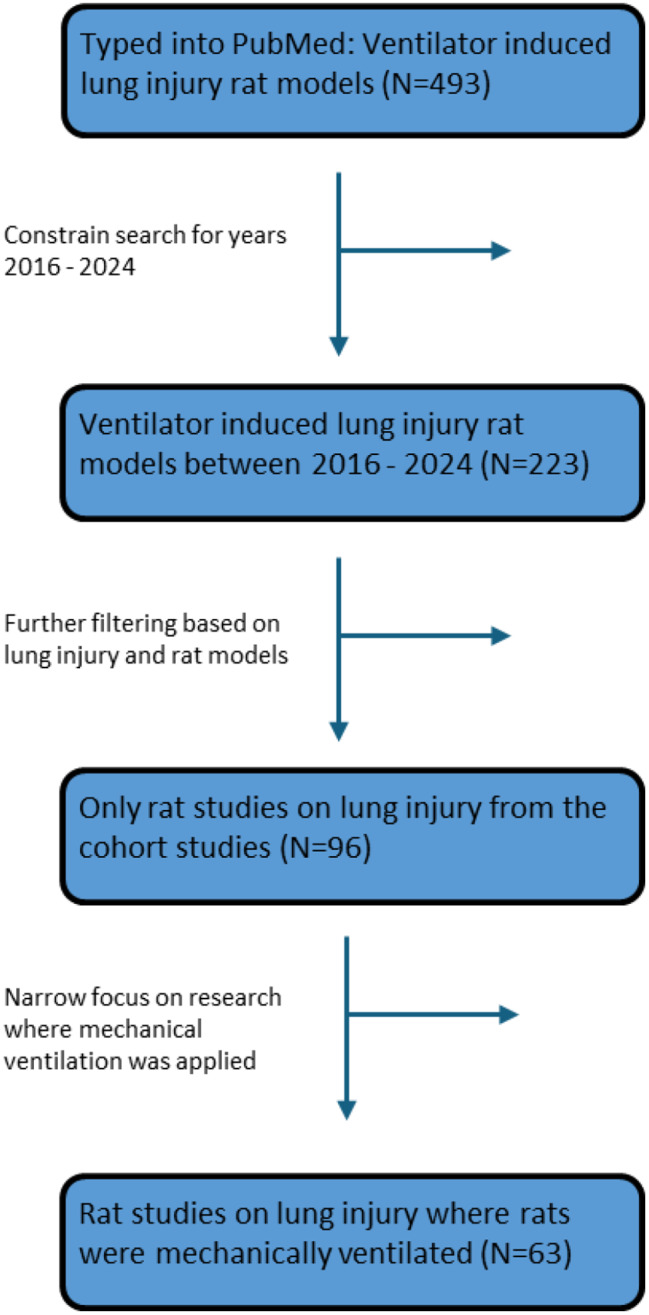
The overall workflow for selecting papers for this review

The overall workflow for selecting papers for this review is seen in Fig. 1.

First, the PubMed enquiry “Ventilator induced lung injury rat models” was typed into the PubMed search tool. Years of publications were set we set for 2016–2024. Abstracts were read and only studies selected where rat models were used and lung injury was induced. The articles were studied and the ones where MV was employed were identified.

After filtering the search, 63 papers were deemed appropriate for this review.

## Rat strains

Choosing the appropriate rodent for research can be critical. Approximately 95% of all animal models in research are mice and rats, with mice taking up about 75% [5]. They are common choices distinctly for their ease of handle, availability, and their analogous physiology and genetics to humans [5]. For research purposes the main divergence between mice and rats are concerned with size, handling, and social and cognitive behaviour. When it comes to various procedures the size of the research animal is important and therefore rats may be preferred over than mice. This is especially true for VILI studies as the connection to the ventilator tubes to the airways in smaller animals can be technically difficult. Also, imaging, harvesting organs and collecting bronchoalveolar lavage fluid (BALF) is made easier. Rats tend to be easier to handle and less stressful than mice, rarely biting handlers unless in extreme pain or distress. Rats are not as territorial as mice and less aggressive in social situations and less prone to stress and anxiety in cognitively challenging situations [5, 69].

The two types of rats utilized in the studies analysed were either Sprague Dawley or Wistar. Of the 63 papers reviewed here, 37 used Sprague Dawley [7, 8, 10, 13–15, 18, 20, 22, 26, 29, 32–35, 39, 40, 42–45, 47–49, 51, 55, 56, 58–61, 63–68] and 24 used Wistar [6, 9, 11, 12, 16, 17, 19, 21, 23–25, 27, 28, 30, 36–38, 41, 46, 50, 52–54, 57], one publication used both [62] and in one case the strain was not indicated [31]. Both breeds are outbred, albino rats. The main differences between the strains are that the Wistar rats are more active, they have shorter tails, wider head and longer ears than the Sprague Dawley, whose popularity can be somewhat attributed to their calm nature and ease of handling [70–72]. No genetic manipulations of the strains were seen in the reviewed papers and all rats used were male. Age of the rats was often indicated as “adult” and the weight range listed. As male Sprague Dawley rats weigh from 50 g at 3 weeks and up to 550 g at week 12 and Wistar rats weigh from 50 g at 3 weeks and up to 500 g at week 12, the age of the animals can quite easily be discerned. Only in 2 studies very young (13–15 days old) rats were used [37, 62].

## Physiological parameters

### Tidal volume/airway pressure

The most common method of expressing the ventilator output is through tidal volume. The pressure needed to reach an indicated tidal volume can change as lung stiffness and compliance changes with increasing lung injury. In all but four studies, the tidal volumes are the indicated factor of mechanical stress. Ergin Özcan et al. studied the effects of different recruitment manoeuvres on bacterial translocation and VILI. They used varied peak inspiratory pressure (PIP) and positive end-expiratory pressure (PEEP) [22]. Petersen et al., in 2016, used PIP of 13–35 cmH_2_O in their ventilation studies [13]. In two consecutive studies, Horie et al. used 35 cmH_2_O of P_insp_ for their studies [14, 32].

There is a clear difference in the articles between those that use a two-hit method (Reviewed in the next chapter) to induce ARDS/VILI and those that only use injurious tidal volumes. Researchers that use injurious tidal volumes tend to use low/protective volumes of approx. 5–10 mL/kg, medium volumes of 12–16 mL/kg and high/injurious volumes of 15–40 mL/kg [16, 17, 21, 22, 29, 31, 34–37, 55, 59, 61, 63, 66, 67]. The high variability in the range of high tidal volume is noticeable.

PEEP is used for the low/protective tidal volumes to avoid collapse of alveoli during expiration, but set at zero for the higher/injurious tidal volumes [20, 21, 44, 55, 66, 67], presumably to produce atelectrauma as well as overdistension.

Breaths per minute (BPM) are modulated according to tidal volume by some researchers [21, 35, 45, 55, 58, 62, 67], but more prevalent is the use of one set BPM. This could be problematic and induce hyper- or hypocapnia in the rats, where the use of minute ventilation (Breaths per minute * Tidal volume) might be a better option. The range of BPM is 35–90 across the articles.

Table 1 summarizes physiological parameters reviewed.

**Table 1:** The figures in the table below are to some extent the authors interpretation of methods used in the reviewed studies as there was a great variability within each parameter. Also, their definition between studies varied. Information of parameters were lacking in some studies

Type of Intubation	Nr. of studies
Surgical tracheostomy	59
Oral endotracheal intubation	1
Not specified	3
**Type of Ventilator**	**Nr. of studies**
Harvard Apparatus	16
Servo	9
Not Specified	11
Other	27
**Tidal Volume/Airway Pressure**	**ml/kg**
Low/Protective	5–10
Medium	12–16
High/Injurious	15–40
**PEEP**	**cmH2O**
Injurious ventilation	0
Range of PEEP in Protective ventilation	0–11
**Respiratory Rate**	**BPM**
Average	54
Range	18–120
**Time of ventilator treatment**	**Hours**
Average	4
Range	< 1–6

### Time of ventilation

Time of ventilation of the rats varies but the most common is 4 h [11, 15–19, 26–30, 34, 35, 40, 43, 45, 46, 48, 49, 52, 56, 57, 59, 63, 68], it ranges, however, from under an hour, up to 6 h. Settings for breaths per minute were from 18 to 120, usually adjusted with tidal volume, where higher tidal volume used resulted in fewer breaths per minute and vice versa. Fraction of inspired oxygen (FiO_2_) was set to the atmospheric level of 21% in most studies that mentioned it but was set at up to 80% in one study [16].

### Intubation

The most common method of intubation of the rats was through a surgical tracheotomy, where a tube was inserted into the trachea, usually a Y-canula, which arms were then connected to the inspiratory and expiratory tubes of a mechanical ventilator unit. Interestingly, only one research stated that they used oral endotracheal intubation [45].

### Ventilators

When noted, the choice of mechanical ventilators that researchers use vary considerably. A popular choice is the Harvard Apparatus (Harvard Apparatus, Massachusetts, USA) brand with 16 of the research papers using their ventilators [8, 10, 18, 19, 21, 24, 30, 31, 33, 35, 53, 58, 60, 61, 63, 64]. The Swedish Servo (Siemens, Solna, Sweden) ventilators were the second most popular choice for researchers with 9 publications using them [12, 13, 22, 23, 25, 36, 38, 41, 51]. Other brands of ventilators were used less frequently, and some labs have made their own ventilators [39, 67].

### Anaesthesia

Anaesthesia was, in almost all cases, administered intraperitoneally (i.p). One study anesthetized the rats by way of continuous intravenous (i.v.) infusion of Thiopental (60 mg/kg/h) followed by paralyzing the animals with a bolus injection of Pancuronium Bromide (1 mg/kg) [55]. Just under half the reviewed papers report using Pentobarbital injection as the main anaesthesia [6, 7, 11, 16, 17, 27–30, 34, 35, 39, 40, 42–47, 52, 56, 57, 60, 61, 65–68]. The doses vary from 25 mg/kg − 80 mg/kg with maintenance at 5–20 mg/kg/h. Pentobarbital is supplemented with Rocuronium at 0.6 mg/kg in three studies [16, 28, 52], Fentanyl at 0.05 mg/kg in three studies [29, 34, 56] and with Xylazine in one study, where they used 10 mg/kg [35]. Ketamine and Xylazine were used together in 18 studies [8, 10, 14, 19–21, 25, 26, 31, 32, 37, 49, 53, 54, 58, 59, 62, 64], with Ketamine administered at a range of 35–100 mg/kg and Xylazine at 2–20 mg/kg. Ketamine was also used together with Midazolam in five studies [23, 24, 36, 38, 50], where Ketamine was administered at 50–100 mg/kg and Midazolam at 2–5 mg/kg. One study reported using only Ketamine at 100 mg/kg [33]. Midazolam was used together with Fentanyl in one study [51] at 5 mg/kg/hour and 10 µg/kg/hour, respectively. Other compounds included 10% Chloral Hydrate at either 1.5 mL/kg or 300 µg/kg [63], Thiopental at 60 mg/kg [55] and Urethane at 1.2 mg/kg [9]. Three studies reported using Isoflurane as the main anesthesia at 1–3% [12, 13, 22], supplemented either by Pentobarbital (60 mg/kg) [13] or Ketamine (50 mg/kg), using Vecuronium Bromide (0.5 mg/kg) for paralysation [22]. Sevoflurane 8% vaporized in a mixture of air and oxygen with an FiO_2_ was used in one study [41], with Sevoflurane at 2.5% and 0.3 mg/kg of Rocuronium administered every 30 min for maintenance. A summary of anaesthetic agents used in the reviewed articles can be found in Table 2.

**Table 2:** General summary of anaesthesia used in the reviewed articles, although supplementation of anaesthesia varied considerably

Anaesthesia			# of Studies
Pentobarbital			21
	Supplemented with:		
	Rocuronium		3
	Fentanyl		3
	Xylazine		1
Ketamine			
	Supplemented with:		
	Xylazine		18
	Midazolam		5
Midazolam + Fentanyl			1
10% Chloral Hydrate			1
Thiopental			1
Urethane			1
Isoflurane (1–3%)			1
	Supplemented with:		
	Pentobarbital		1
	Ketamine		1
Sevoflurane 8% Vaporized			1

## Injury models

In order to capture the complexities of ARDS and VILI within a short time period, it often does not suffice to only mechanically challenge the animals with a ventilator. A two-hit model approach can be more appropriate, and in 27 of the papers reviewed here, the researchers opted for this type of model system. However, the methods for inducing this two-hit injury, vary. The challenges in modelling animal lung injury and their relevance to clinical reality has been reviewed elsewhere in recent papers [73, 74], while the difference in approaches is exemplified here.

### Intratracheal hydrochloric acid

One of the most utilized compounds to induce lung injury in rats is Hydrochloric Acid (HCl) instilled intratracheally, but in various doses, pH and time. HCl is allowed to cause injury followed by various different ventilator settings. Some papers cite aspiration of gastric contents, a serious complication associated with general anaesthesia and often leads to ARDS with high mortality rate [75, 76], as a reasoning for this model system. Drachman et al., ventilated their rats with 12 mL/kg tidal volume with a PEEP of 3 cmH_2_O for 3 h. After 70 min they cause lung injury in the rats using a 2.0 mL/kg tracheal instillation of pH 1.25 HCl [39]. Fanelli et al. induced injury by intratracheal instillation of 2.5 mL/kg of lactated Ringer´s solution, titrated to pH 1.0 with HCl. This dose was based on their pilot study results of a significant decrease in the ratio of PaO_2_:FiO_2_. Rats were then ventilated for 3 h with Vt of 6 mL/kg and PEEP of 8 cmH_2_O [51]. In their 2016 paper, Cereda et al. used intratracheal induction of HCl at 2.5 mL/kg at pH 2.15 before ventilating the rats at Vt 6 mL/kg with a PEEP of 10 cmH_2_O for 1 h. After stabilization they continued ventilating the animals with moderate Vt of 12 mL/kg and 3 cmH_2_O PEEP for 3 h [67]. A study from the same group came out in 2017, using similar HCl induction methods, but in this study the animals received ranges of HCl doses (1–4 mL/kg), were ventilated at a protective Vt of 6 mL/kg, with PEEP at 10 cmH_2_O for one hour, and non-protective ventilation at Vt 12 mL/kg at 5 cmH_2_O, for 4 h (or until death) [65]. In a 2018 study, this group also used HCl for induction of lung injury, using 2.5 mL/kg of HCl at pH 1.25 through the endotracheal tube. Rats were then ventilated at Vt 6 mL/kg with 10 cmH_2_O PEEP and at Vt 12 mL/kg with 3 cmH_2_O PEEP, for up to 3 h [66]. Henzler et al. [40] refer to methods of their previous paper [77], where 5 mL of HCl solution (pH 1.0) was instilled via the tracheal cannula and then removed after 5–10 s. This was followed by ventilation of Vt 8 mL/kg with PEEP set to 5 cmH_2_O for up to 4 h. This same group published in 2022, where they induced lung injury by endotracheal instillation of 2.5 mL/kg of unbuffered HCl (pH 1.25). Injury was allowed to develop for 1 h under Vt 8 mL/kg ventilation with 5 cmH_2_O PEEP. Animals were then ventilated in groups with varying Vt (8–12 mL/kg), respiratory rate and added CO_2_ to maintain normocapnia or induce hypercapnia [43].

### Surfactant deactivation

Kollisch-Singule et al. published a paper in 2016, where the group used 0.2% of polysorbate 20 in normal saline (5 mL/kg) to induce surfactant deactivation. Then rats were ventilated at Vt 16 mL/kg with 0 cmH_2_O PEEP for 10 min. This model was set up to mimic a patient with a direct pulmonary insult, such as pneumonia, aspiration or inhalation injury [20]. Mingote et al. used a method of inducing ARDS introduced in the late nineties [78], where repeated bronchoalveolar lavages (BAL) are employed. They performed 5–7 BAL with 10 mL/kg of warmed saline solution 0.9% [41]. This group set individual PEEP for each animal through a complex series of incremental pressure increases. Then they calculated for each animal their individual driving pressure (DP = peak inspiratory pressure – end expiratory pressure, adjusted to pulmonary elastance). In the study, animals were ventilated with DP of 14 cmH_2_O (With Vt ≤ 6mL/kg) and individualized PEEP for 2 h.

### Endotoxins

Exposing rats to the endotoxin LPS induces an inflammatory response with accumulation of mediators and inflammatory cells that mimics the early phase of ARDS [79]. This has been exploited by several of the research covered in this review. In this section, we review the types of endotoxins used, how they were administered, at what concentrations and in brief the ventilator protocols that followed the insult.

Gao and Ju used 500 µg/kg of *E. coli* endotoxin (Escherichia coli endotoxin, 0111:B4, Sigma, St. Louis, MO), administered by intravenous injection, after 30 min, the rats were ventilated at Vt 30 mL/kg for 4 h [11]. Ergin Özcan et al. had a different approach, where they prepared a tube containing 1 × 10^5^ cfu/mL of *Pseudomonas aeruginosa* for each rat, 500 µL of which was instilled through a tracheostomy tube along with 5 mL of air to ensure distribution of the bacteria through the lungs. Rats were then ventilated at various settings as the study concerned different recruitment manoeuvres [22]. In their 2016 paper, Bianchi et al. used *E. coli* derived LPS (LPS serotype 055:B5, Sigma, Israel) and administered it intraperitoneally at 5 mg/kg dissolved in 0.5 mL of 0.9% saline solution. Then, 24 h later, the rats were ventilated at 6 mL/kg with a PEEP of 5 cmH_2_O [24]. In another study from 2016, Kuethe et al. intratracheally instilled rats with *Pseudomonas* LPS (LPS, Sigma, St. Louis, MO) at 1 or 2 mg/mL, dissolved in 0.75 mL of PBS. To allow a full inflammatory response, the rats were left for 48 h and then tracheotomized and ventilated at either protective Vt of 11–19 mL/kg with 2 cmH_2_O PEEP or injurious ventilation at Vt 25–38 mL/kg with 0 cmH_2_O PEEP. Here, the rats were ventilated for up to 15 h under monitoring [44]. Santos et al. administered LPS from *E. coli* (LPS serotype O55:B5, Sigma, St. Louis, MO) to rats, intraperitoneally at a dose of 1 µg/µL of saline solution. This induced mild acute lung injury (ALI). Animals were left for 24 h and then tracheotomized and ventilated at Vt of 6 mL/kg with 5 cmH_2_O PEEP for 1 h [23]. In 2017, Meng et al. published an article where they administered the rats *E. coli* derived LPS (LPS serotype 0111:B4, Sigma, Saint Louis, MO) intravenously at 500 µg/kg. Rats were then ventilated at Vt 20 mL/kg for 4 h, presumably at 0 cmH_2_O PEEP [52]. Moraes et al. installed intratracheally intubated rats *E. coli* derived LPS (LPS serotype 055:B5, Ultrapure, Invivogen) at 1 µg/µL. After 24 h rats were tracheotomized and ventilated at 6 mL/kg, 3 cmH_2_O PEEP, to maintain normocapnia. The rats were ventilated at three different tidal volumes, Vt 6 mL/kg, Vt 13 mL/kg and Vt 22 mL/kg for 2 h [36]. Ju et al. induced ARDS by injecting rats intravenously with 500 µg/kg of *E. coli* LPS (LPS serotype 0111:B4, Sigma). Thirty minutes later the rats were subjected to ventilation for 4 h at Vt 17 mL/kg [27]. The same group publish another paper in 2018 where rats again received 500 µg/kg of *E. coli* LPS (LPS serotype 0111:B4, Sigma) by intravenous injection. As before, 30 min later the rats were ventilated for 4 h, but at a higher Vt of 30 mL/kg [16]. Elder et al. state that they intratracheally instilled *E. coli* LPS (LPS serotype O55:B5, Sigma, MO) at 2 mg/kg in saline. Then the rats were ventilated for 2 h at either non-injurious Vt of 7 mL/kg and 2 cmH_2_O PEEP or injurious Vt 25 mL/kg with 0 cmH_2_O PEEP [55]. In 2020, Rocco et al. examined unanalysed data from a previous study [80] where rats were intratracheally instilled with *E. coli* LPS (LPS serotype O55:B5) at 20 µg/µL in saline solution. Twenty-four hours later, rats were ventilated at varying Vt, ranging from 6 to 22 mL/kg, with PEEP ranging from 3 to 11 cmH_2_O, for 1 h [25]. In their paper from 2021, Cruz et al. intratracheally instilled rats with 5 × 10^7^ cfu of *Pseudomonas aeruginosa* (ATCC27853, FIOCRUZ Bacterial Culture Collection) bacterial culture. Then, 24 h later, rats were ventilated for 1 h in various conditions, one of them with Vt of 6 mL/kg [38]. Jiang et al. inserted a 16 G catheter in the trachea orally and instilled with 50 mg/mL LPS (Origin and serotype unknown) and left for 30 min. Then rats underwent tracheotomy and ventilation for 4 h at either low Vt of 6 mL/kg and 5 cmH_2_O PEEP or Vt 30 mL/kg and 0 cmH_2_O PEEP [46]. In a recent study, Fernandes et al. intratracheally installed rats with *E. coli* (Origin and serotype unknown) LPS at 9.6 × 10^6^ U/mL diluted in 200 µL of saline. Twenty-four hours later rats were ventilated with Vt 6 mL/kg and PEEP of 3 cmH_2_O for 2 h, but as this was a study on increases in PEEP or recruitment manoeuvres, for groups of rats, the PEEP was increased within the 2 h [50]. Lastly, Bermudez et al. intratracheally instilled rats with *E. coli* LPS (LPS serotype 0127:B8) at 0.1 mg/kg. Then, 18 h post instillation, rats were ventilated at Vt 20 mL/kg with 0 cmH_2_O PEEP for 4 h [26].

### Elastase induced emphysema model

In order to induce emphysema in their rat model, Henriques et al. intratracheally administered porcine pancreatic elastase at 2 IU in 0.1 mL of saline solution (Sigma Chemical Co, St. Louis, MO), once weekly for 4 weeks. Then rats were ventilated using Vt 6 mL/kg and PEEP 3 cmH_2_O [12]. Some variation was used in the ventilation protocols as the aim of the study was to compare between variable and conventional volume-controlled ventilation.

## American thoracic society key domains of experimental acute lung injury

**Fig. 2:**
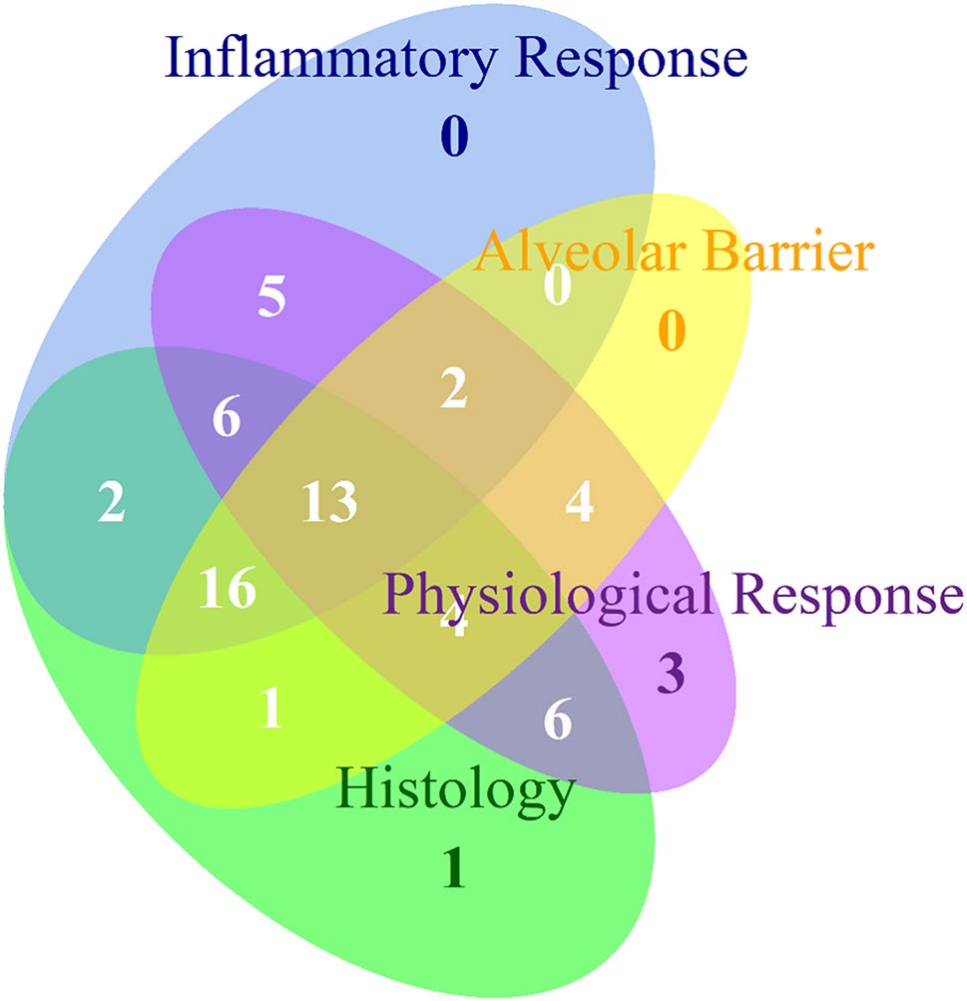
Venn diagram of the four domains of acute lung injury and how they are represented in the articles reviewed

In the updated version of the American Thoracic Society´ features and measurements of experimental acute lung injury in animals [81], the four domains reflecting the key pathophysiologic features and underlying biology of experimental acute lung injury are the following: Histological evidence of injury, alteration of the alveolar-capillary barrier, presence of an inflammatory response and evidence of physiological dysfunction. In this section we have summarized if the articles reviewed here recapitulate features of these domains. This can be seen in Fig. 2.

In the reviewed articles, histological evidence of injury is most often seen in hemotoxilin/eosin stainings. These images often reflect the extent of the injury and often show some attenuation of injury through a tested drug, inhibitors/activators or other means. These images often reveal.

an infiltration of neutrophils as a result of the injury.

Alteration in the alveolar-capillary barrier is often shown by way of alterations in total protein in BAL, where an increase in the amount of proteins detected reflects barrier failure. Other applied methods include Evan´s blue dyeing and measuring the wet/dry ratio of the lungs. Increased inflammatory response is very often characterized by an increase in cytokine detection, most notably IL1β, IL6 and TNFα. An increase in neutrophil activity, measured by NE or MPO assays, is also represented. Evidence of physiological dysfunction can, for instance, be seen in alterations in blood gas measurements (PaO_2_/FiO_2_), respiratory rate and lung compliance/elastance. As can be seen in Figs. 2 and 13 of the 63 articles explore or document all four of the features and just over half of them (32) having at least three. Two of the features are represented in 14 articles and one feature is represented in four articles.

## Conclusions

The aim of this review was to showcase important methodological features of mechanical ventilation research in rats over the last few years, mainly to explore how different or similar the methods are that researchers have been using to mimic VILI or ARDS. The research methods reviewed here are often means to different goals and can be difficult to compare fairly, which is why we highlight the methodological parameters in what we consider to be an unbiased way. There are important similarities between the studies and important differences. Similarities are most notable in rat strains used, intubation methods, ventilation time and FiO_2_. Only two rat strains were used, and no genetic manipulations were performed. Only one research used oral intubation for connecting the airways to the mechanical ventilator and ventilator time was relatively short, about 4 h. Atmospheric air was most often used for ventilation.

The type of anaesthesia varied though, and rather few studies stated use of muscle relaxation which might affect the extent of lung injury. Mechanical strain considered to cause VILI was created with substantially different settings of PEEP, PIP, Vt and BPM. Methods to calculate mechanical power to estimate the forces the lungs are subjected to, with the various ventilatory settings used, might ease comparisons between studies [82].

Also, in the two hit models the methods used to cause injury to the alveolar capillary membrane vary substantially. From surfactant deactivation to direct injury to the lung epithelium either through chemical or inflammatory insult through tracheal installation and to indirect injury through inflammatory mechanisms of different types, given intravenously or intraperitoneally, possibly primarily affecting the endothelium initially. Other differences were seen when comparing studies that involve testing a drug candidate for VILI/ARDS versus basic research, where the researchers were interested in using the animal model to increase basic understanding of VILI/ARDS.

These different aspects of VILI/ARDS can create a dilemma for researchers trying to recapitulate clinical reality. As can be seen in the 2-hit models of ALI in the reviewed articles, researchers use a variety of methods to induce ARDS. HCl injury can mimic the aspiration of the gastric contents into the lungs, which can be a direct injury to the lung itself but is also mediated by neutrophil dependent mechanisms [75, 76]. This type of injury can reflect mechanical injury to the lung, but also the following inflammatory response. Risk of fatally injuring the rats makes this method challenging [74]. The use of Endotoxins emulates the serious adverse effects of conditions such as pneumonia, sepsis and the possible ensuing ARDS. Studies either use Endotoxins directly or introduce cultured bacteria into the lungs of the rats. This type of injury mimics the biotrauma aspect of VIL, but can have limited systemic effects [74]. Another type of model researchers use is to deplete surfactants from the alveoli, either by frequent BALF washings or by way of polysorbate. This models the atelectrauma aspect of VILI, whereas the absence of surfactants leads to the collapse of the alveoli which in turn can also lead to ARDS.

According to the American Thoracic Society workshop report, there is significant variability in what researchers consider ALI in an animal model system [81]. The summary of the 50 participants in the workshop was that ALI would be characterized as a “multidimensional entity” that recapitulates the four key pathophysiologic features of ALI and ARDS. These four features were (1) histological evidence of tissue injury, (2) alteration of the alveolar-capillary barrier, (3) presence of an inflammation response, and (4) physiologic dysfunction. Each of these features have listed intrinsic measurements. In this review, we categorized the articles based on how well they represented each of the four features based on the measurements within each feature (Fig. 1). The suggestion of the workshop was that although mechanistic studies could focus on one feature of Ali, the model must document alterations of at least three of the four domains to qualify as experimental ALI [81]. In 45 of the 63 articles reviewed here, there were documented or explored at least 3 of the domains, with only four of the articles documenting only one aspect. Of course, each of the four features of experimental ALI have many relevant measurements which can be difficult to quantify, but researchers clearly aspire to represent ALI to the best of their abilities.

Animal models have enhanced our understanding of complex mechanism and pathophysiology of VILI [73]. Methods used to study VILI in rat models vary markedly and differ from being generated only with mechanical strain of different amounts to using two hit models with various means of inducing a clinical state mimicking direct or indirect cause of ARDS, adding mechanical strain afterwards. It also varies how well the guidelines from the American Thoracic Society are followed to describe the injury. All this causes an uncertainty of how to interpret and compare results, but it is to be expected that reaction to different possible therapeutics will vary between different models.

A general limitation of this study lies in the inherent difficulty of comparing studies with such a variety of parameters. This makes any recommendation or summary of the methods impossible. As such, this paper rather highlights the differences and the lack of a consensus.

In summary, methods to induce VILI/ARDS in rat models vary significantly, which reduces efficiency when translating results from in vivo settings to the clinic. Hopefully this review will give researchers insights into recent VILI/ARDS rat study methods. Perhaps a more standardised approach should be applied but that would require formation of a consensus among researchers within the field.

## Data Availability

All articles reviewed here were found using a PubMed search with the search title “ventilator induced lung injury rat models”, filtering the publication year from 2016 to 2024. From the 493 matching hits, we concentrated on models where rats were mechanically ventilated, leaving a pool of 63 publications.

## Abbreviations

ARDS: Acute respiratory distress syndrome
BALF: Bronchoalveolar lavage fuid
BPM: Breaths per minute
FIO2: Fraction of inspired oxygen
ICU: Intensive care unit
MV: Mechanical ventilation
PEEP: Positive end-expiratory pressure
PIP: Peak inspiratory pressure
VILI: Ventilator-induced lung injury
Vt: Tidal volume
